# A Brief Journey into the History of and Future Sources and Uses of Fatty Acids

**DOI:** 10.3389/fnut.2021.570401

**Published:** 2021-07-20

**Authors:** Michela Cerone, Terry K. Smith

**Affiliations:** Biomedical Sciences Research Complex, University of St Andrews, St Andrews, United Kingdom

**Keywords:** Cell-factory, polyunsaturated fatty acids, biofuels, biotechnology, microalgae, plants, oleaginous microorganisms, sustainable sources

## Abstract

Fats and lipids have always had a primary role in the history of humankind, from ancient civilisations to the modern and contemporary time, going from domestic and cosmetic uses, to the first medical applications and later to the large-scale industrial uses for food, pharmaceutical, cosmetics, and biofuel production. Sources and uses of those have changed during time following the development of chemical sciences and industrial technological advances. Plants, fish, and animal fats have represented the primary source of lipids and fats for century. Nowadays, the use of fatty acid sources has taken a turn: industries are mainly interested in polyunsaturated fatty acids (PUFAs), which have beneficial properties in human health; and also, for high-value fatty acids product for innovative and green production of biofuel and feedstocks. Thus, the constant increase in demand of fatty acids, the fact that marine and vegetable sources are not adequate to meet the high level of fatty acids required worldwide and climate change, have determined the necessity of the search for renewable and sustainable sources for fatty acids. Biotechnological advances and bioengineering have started looking at the genetic modification of algae, bacteria, yeasts, seeds, and plants to develop cell factory able to produce high value fatty acid products in a renewable and sustainable manner. This innovative approach applied to FA industry is a peculiar example of how biotechnology can serve as a powerful mean to drive the production of high value fatty acid derivatives on the concept of circular bioeconomy, based on the reutilisation of organic resources for alternative and sustainable productive patterns that are environmentally friendly.

## Introduction

**Figure 1:**
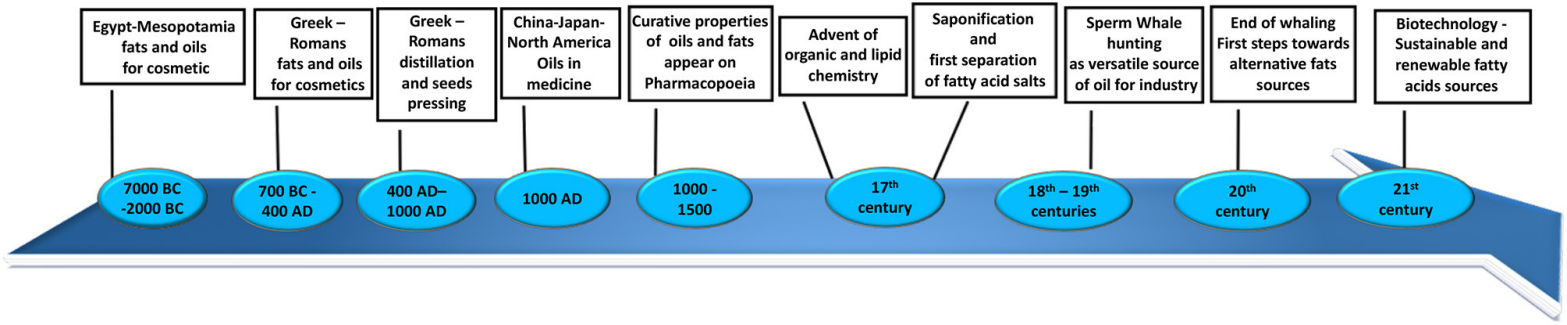
Timeline of fatty acid source discovery, applications, and advances throughout history.

Throughout the history of humankind, fats and lipids have been considered extremely important because of their value in food, cosmetics, and natural medicine, as well as many other domestic applications (such as cooking and candle wax). The first recorded usage of vegetable oils and animal fats dates back to Mesopotamia (7,000 BC) (Figure 1) and ancient Egypt (5,000 BC) (Figure 1) (1). They were used for cosmetic applications such as body oils and lotions. Around 2,000 BC (Figure 1), these populations started producing scented oils for mummification and for personal hygiene, healthcare, and cosmetics. They introduced a new technique based on maceration in oils of flowers, leaves, spices, resins, and in some cases, pigments (1). Contemporary documents describe a surprisingly broad number of sources of oils, fats, and waxes. From very common seeds such as linseed and poppy seeds, to indigenous trees like cedar and palm, fruits such as olives and avocados, fish and even some remarkable animal oils such as hippopotamus or crocodile oils, it is clear that even in more primitive ages the knowledge of lipids was more developed than we assume (1). This tradition was followed by the great Mediterranean societies of the ancient Greeks and Romans (Figure 1). They introduced new techniques for production of oils and lotions such as distillation and seed pressing (2). Between 400 and 1,000 AD (Figure 1), despite the world being preoccupied by the Dark Ages, in some part of Europe and in China, Japan, and North America, great advances were made in the usage of oils and fats especially applied to medicine and alchemy (3, 4). During the Middle Ages, curative properties started to be attributed to oils and fats and were documented in the Mediaeval and seventeenth centuries European Pharmacopoeias (Figure 1) (5).

With the advent of organic and lipid chemistry and the industrial revolution, production methods used changed radically and started to move toward large-scale production (6). An example of the first innovative process introduced on a large scale between the seventeenth and eighteenth centuries was saponification (Figure 1). The basic process for making soap known so far was to boil animal fats or oils with a strong alkali, with incorporation of salts to then separate fatty acid salts and the glycerol from the final mixture. During the second half of the eighteenth century, the production of soaps increased to become an important industrial scale operation (Figure 1) (6). During the eighteenth and nineteenth centuries, the understanding of biochemical properties and applications of fats grew hand-in-hand with chemistry as a whole. Thus, innovation and broader chemical knowledge gradually resulted in the development of novel industrial applications and uses for fatty acids and, consequently, the constant search for new and varied sources of those in nature (2). It was then that men started hunting a very rich source of fats, an “industry of oils at its day” as it was later defined: sperm whales (Figure 1) (7). They were the largest toothed whales, known at the time for their huge head filled entirely with a peculiar waxy substance called spermaceti (7). This very precious oil comes from the blubber and acoustic fat bodies, which we now know to be essential for signal transmission in whales (8). The versatility and the large quantities of the spermaceti oil were appealing to the fats industry at the beginning of nineteenth century: the liquid form was used to fuel lamps and the congealed form was used for candles, soaps, and cosmetics (7). The whaling industry saw a decline from 1880 until 1925, to then increase again during the World War II: by the end of 1958, more than 20,000 sperm whales were killed every year, and their waxy oil was used to produce cattle fodder, dog food, vitamins, supplements, glue, leather preservatives, and brake fluids (Figure 1) (7). The number of whales decreased so drastically that in 1982 whaling was declared illegal. Since the start of whaling, the population of sperm whales has decreased from around one million to just few hundred thousand. This resulted in an incredible loss for the marine ecosystem, because of the importance that whales have in producing phytoplankton, which recycle CO_2_ from the atmosphere (9).

Nowadays, the use of fatty acid sources has taken a turn from the past: food, cosmetics, and pharmaceutical industries are mainly interested in polyunsaturated fatty acids (PUFAs), which have been shown to possess beneficial properties in human health (10). Fatty acids have also been recently valued as innovative and green source for production of biofuel and feedstock (Figure 1) (11, 12). PUFAs are mainly produced by marine phytoplankton and contained in fish and seafood, but climate change has dramatically affected the marine ecosystem. This is because of the high level of carbon dioxide emission and ultraviolet (UV) irradiation, both of which have resulted in a decrease in growth marine sources and reduced synthesis of PUFAs (13). Vegetable oils cannot account for the current shortfall, and the cost of microbial production is too high (14, 15). Fish and vegetable oil sources are not adequate to meet industrial needs, so research in this area has started looking at the genetic modification of algae, bacteria, yeasts, seeds, and plants as bioengineered factories to produce PUFAs in a larger amount corresponding to the increased world demand (16).

## The Discovery and Early Chemical Knowledge of Fatty Acid Molecules

At the end of the eighteenth century, Antoine Lavoisier was the first scientist to determine the elemental composition of fats and oils. He established that fats and oils consist mainly of carbon and hydrogen atoms; starch and sugars instead contain also oxygen atoms; therefore, he considered the last ones as oxides of fats (2). One of the most important figures in fatty acid chemistry at the time was Michel-Eugene Chevreul, who brought great development into the understanding of natural fats, when organic chemistry was far behind the modern technologies we are used to today. He started his investigation on animal fats, which led to the first class of naturally occurring organic substances to be chemically studied and understood. He was the first chemist able to isolate a crystalline material with acidic properties by treating soap obtained from animal fats with acid: a fatty acid molecule (2). The first isolation of a fatty acid was followed by isolation and studies of many other fatty acids from butyric to stearic. He defined saponification as the chemical process by which fatty acids and glycerol are obtained *via* a process described as fixation of water and displacement of glycerol by alkali to give fatty acids. Chevreul et al. (17) was also able to experiment and introduce techniques for the isolation of fatty acids based upon their fractional solubility in several solvents, multiple crystallisations, and determination of their purity by measures of melting points, introduced now for the first time (17). In 1854, Marcellin Berthelot expanded the studies of Chevreul, focusing on the synthesis of fats using glycerol and fatty acids (2). This was the first time that an organic substance that does not occur in nature was synthesised in a laboratory. Around the same time, the first study on fatty acid metabolism was carried out by von Liebig (18). This study was based on the idea that quantitative analysis of organic molecules would give information on biochemical transformation in nature, and therefore also in human body through the addition and removal of food sources, gases, liquids, and excretion products. He elaborated a general equation to explain how sugars are converted to fats in the human body. This equation put the foundation for studies of metabolic reactions but did not confirm his theory that fats are formed solely from sugars (2, 19). Later in the nineteenth century, Felix Hoppe-Seyler discovered the phospholipid lecithin (2, 18), while Johann Ludwig Wilhelm Thudichum described the chemical composition of the brain fat as we still know it today (2, 11). At the beginning of the twentieth century, fats were considered a good source of energy and fat-soluble vitamins, but they were not recognised to be essential because scientists believed that they could all be synthesised from dietary carbohydrates (20). In 1929, George and Mildred Burr reported a study in which they showed that lack of dietary fatty acids led rats to develop a deficiency disease, concluding that fatty acids are essential nutrients (20). In particular, they confirmed that linoleic acid was essential because its presence in the diet of rats would prevent the disease. They later defined the concept of omega-3 linolenic acid as another essential fatty acid analogous of linoleic acid. These findings set a great change into the chemistry of lipids, and they are considered a landmark for the lipidomic research until today (20).

## Fatty Acids in the Twenty-First Century: Value, Production, and Consumption Worldwide

How has the interest in fatty acids changed in the contemporary time? Has it developed together with the modern society? If so, how? From the advent of the industrial revolution until the twentieth century, the yield and stability, and the quality and form of fatty acid products were radically improved by the introduction of new techniques for fat extraction, the process of refrigeration, the addition of preservatives and antioxidants, and by the hydrogenation of unsaturated fatty acids. These aspects had a great impact on the evolution of fats industry and market, and consequently on human usage. Saturated fats became cheaper and easily accessible for the population in the form of butter, shortenings, margarine, therefore increasing the daily dietary intake of saturated fatty acids and changing radically the western diet (21). With the change in human diet during the contemporary era, modern diet has diverted from one very rich with omega-3 PUFA and well-balanced ratio of omega-6/omega-3 (1:1), to one rich with saturated fatty acids and omega-6 instead of omega-3 (22). This unbalance has shown to be harmful for human health and one of the possible causes of chronic diseases (23). Therefore, the interest in increasing the intake of PUFAs in the human diet has grown rapidly in the food industry (24). Nowadays, fats and oils are largely produced and consumed worldwide at different rates, relying primarily on vegetable sources (24). The greater production is driven by Asia, which accounted in 2018 for more than half of the world global production of fats in the form of soybean, canola, and palm oils (24). Asia is also the greatest consumer of fats and oil in the world, led by China and India, and the United States is second. Palm oil represents 30% of the vegetable oil consumed followed by soybeans oil. Animal fats still account for a large part of the total consumption, but it has not seen an increase in recent years, staying steady, because of health concerns leading toward higher consumption of high-value and essential omega-3 PUFAs. Overall, the world fat consumption for food usage is increasing to a rate of around 3% every year, and the demand for biodiesel production from fats and oils is also growing fast (24). The interest in fats has changed and developed toward the so defined “food-energy-water nexus”: fat chemistry and technology represent a potential platform to highlight synergistic approaches in search for new sources for the main components for food and energy production based upon renewability, respect of the ecosystem, and circular bioeconomy (25). This is certainly one of the biggest challenges for science and industry of our time, of which fatty acid chemistry and technology are a small but exemplar showcase.

## Fatty Acids in Plants: From Natural to Bioengineered Sources

**Figure 2:**
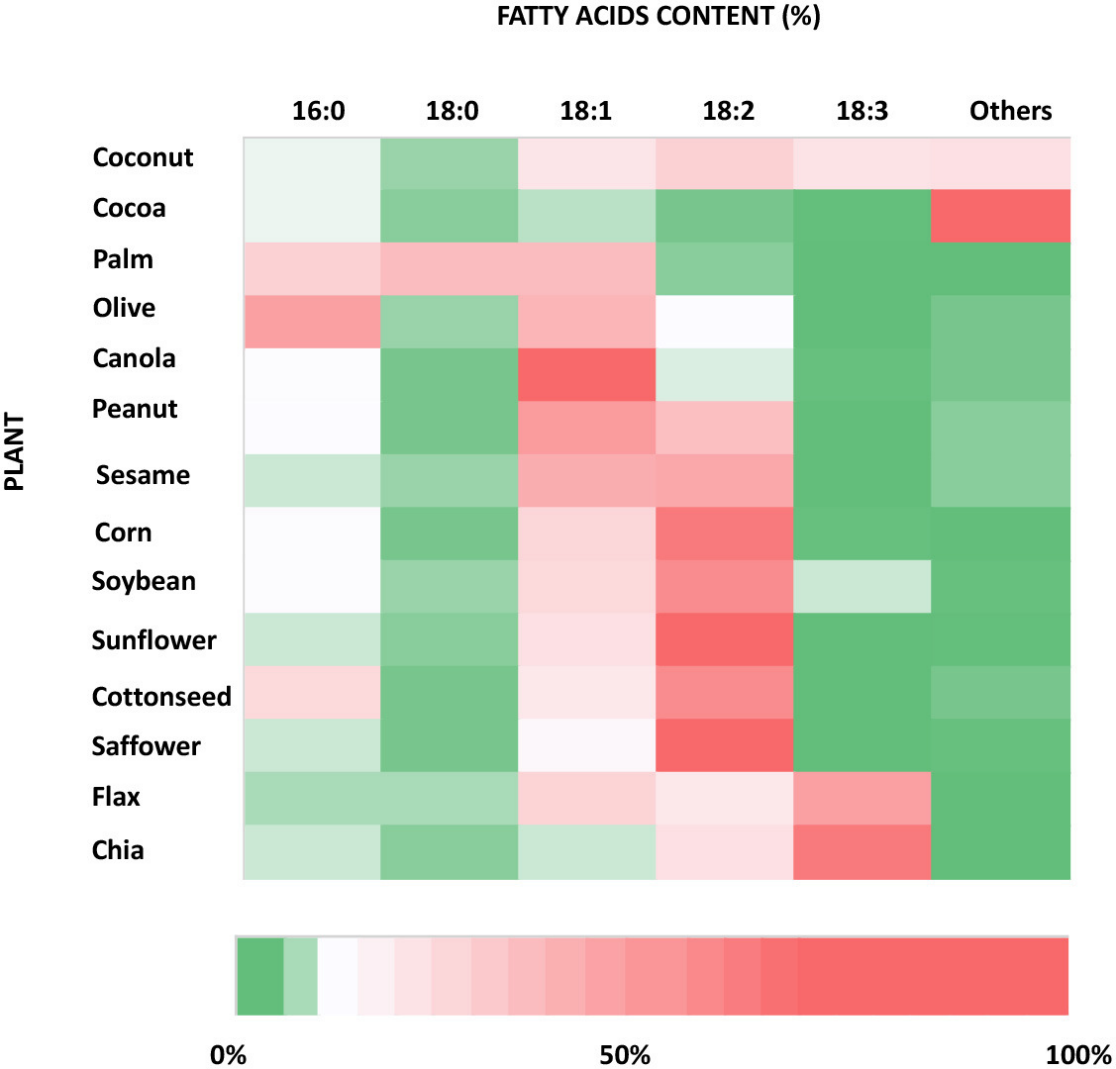
Heat map of the fatty acid content of the most used species of oil producing plants. The information used to produce this graph has been taken from https://lipidlibrary.aocs.org/chemistry/physics/plant-lipid/production-of-unusual-fatty-acids-in-plants.

Plants are rich in fatty acids such as palmitic (C16:0) (PA), stearic (C18:0) (SA), oleic (18:1) (OA), and linoleic (18:2) (LA) acids, which are found in different percentages from species to species of oil plants (Figure 2). They are also considered one of the most important sources of the omega-3 fatty acid, alphalinolenic acids (18:3) (ALA), which is mostly stored in leaves and oleaginous seeds. ALA is present in very high concentrations especially in walnuts, flax seeds, and canola oils and leafy green plants (26) (Figure 2). Vegetable oils and fats have always been the primary sources, earlier through direct consumption, and later through extraction processes, of these classes of fatty acids for food applications.

Thus, plants have been considered a fundamental source to synthesise ω-3PUFAs starting from ALA, in which plants are very rich, to make eicosapentaenoic acid (C20:5) (EPA) and docosahexaenoic acid (n3-C22:6) (DHA), by bioengineering of plant metabolism (27). The very first modification to the fatty acid synthesis in plant (around the 90s) was achieved *via* genetic manipulation of the genes encoding for the enzymes involved in the fatty acid synthetic pathway. Knowing that palmitic acid is very abundant in oils of most of plants, one of the first strategies resulted in increase in the catalytic activity of the enzyme that elongates palmitic acid to stearic. Another method accounted for the reduction of the activity of acyl-ACP thioesterase. The final approach, still in use, consists of the increase in desaturases activity (28). Proceeding with the investigation in this innovative field, progress has been made with the use of heterologous enzymes or newly produced enzymes expressed in plants, to try and mimic the very long chain polyunsaturated fatty acid (VLC-PUFA) synthesis that mostly happens in seeds. This technique has been largely implemented with use of PUFA synthetic enzymes from bacteria, algae, and yeasts (29). Due to the complexity of the biochemical process, many studies have been carried out to reproduce the ability of microalgae enzymes by building an enzymatic machinery able to synthesise VLC-PUFAs in oilseeds in a more efficient manner (30). In this regard, one of the studies that is worth mentioning is the co-overexpression of three different desaturases: Δ9-desaturase from *Isochrysis galbana* (haptophyte), Δ8-desaturase from *Euglena gracilis* (single-celled alga), and Δ5-desaturase from *Mortierella alpina* (oleaginous fungus) into *Arabidopsis thaliana*, also known as thale-cress: it is easy to genetically manipulate because of its relatively small genome and short life cycle. This approach led to the production of EPA and arachidonic acid (ARA) in leaf tissues of plants in very high amounts (31). One of the most common approaches to make VLC-PUFAs is based upon the bioengineering of the pathway that starts off with Δ6-desaturase, which introduces a double bond in position Δ6 of the carbon chain of ALA or LA, followed by elongation of two carbon units, and then by a second process of desaturation by Δ5 desaturases, resulting in the synthesis of ARA and DHA (32). This approach was proved to be successful *via* the insertion of those genes from *Marchantia polymorpha* into tobacco plants (33). A significant achievement in plant biotechnology for production of ω-3 VLC-PUFAs has been reached by the bioengineering of *Camelina sativa*, an oilseed plant that is very cheap and easy to grow. The seeds of this plant can normally produce up to 28% of ALA and 19% of LA, which makes *C. sativa* a very good candidate for the synthesis of ω-3 VLC-PUFAs (34). On those bases, *C. sativa* has been used to introduce a transgenic Δ6-desaturase pathway to convert OA, LA, and ALA into DHA and EPA (35). In particular, it was possible to obtain a fish-like production of DHA by up to 12% by a stable multi-gene construct design and the expression of Δ6-desaturase, Δ6-elongase, and Δ5-elongase from both yeast and microalgae, which showed to be very efficient and industrially relevant in increasing the production of ω-3 VLC-PUFAs at lower cost and higher yield (35). The amounts of VLC-PUFAs produced using these genetic manipulations have been very promising and have suggested that the use of transgenic plant could be one of the most resourceful techniques to increase the production of PUFAs required today (30).

Together with usual FAs from plants described previously, some unusual fatty acid structures have been also found in oilseeds: these can vary in length (short or long carbon chains) and present various functional groups such as hydroxy, epoxy, acetylenic, and cyclopropane. They have been found to have a great potential for non-food industrial uses of fatty acids because of their physical and chemical properties (36). One of the examples is erucic acid (C22:1) produced in large amounts in the seeds of *Brassica napus* and found to be the precursor of erucamide, which is widely used in the production of plastic films and nylon (37). Another example is lauric acid (C12:0), primarily extracted from palm trees, whose surfactant properties have found great applications in the soaps and detergent industry. The problem with these sources is the high cost of production, which could have not overcome the same disadvantages linked to the current use of petroleum sources for production of plastics (28). Thus, once more, genetic manipulation became the key attempt to engineer the production of lauric acid into domestic crops, particularly *via* the overexpression of ACP-thioesterase enzymes, in order to interrupt the elongation of 12 carbon chain fatty acids. An experiment has been successful on *Umbellularia californica*, a bay tree indigenous in California, and also on *Arabidopsis* plants (38). This genetic manipulation has allowed the increase in production of lauric acid over 40%, providing a more sustainable alternative source to imported oilseeds and petroleum derivatives (28).

Furthermore, it is important to highlight at this point the potential that bioengineered plants have as an alternative, sustainable and renewable source for energy and chemical feedstocks for oil industry compared with the traditional oil refining. An alternative to produce cost-effective biofuel from plants, it is to avoid the use of virgin oil trees, which are very expensive, and instead prefer their waste feedstock or their used oils (such as used cooking oil). Furthermore, the optimization in the separation of free fatty acids, which are the starting material to obtain biodiesel, from glycerol, water, and acid or base catalyst eventually used it is very important. This could significantly reduce the presence of contaminants and formation of foam or emulsions, therefore increasing the yield and purity of the final product (39, 40). In order to achieve this goal, lipases and phosphatases have been recently discovered to be particularly efficient. The use of these two enzymes' activities combined and the possibility of those to be immobilised for potential large-scale biofuel production have shown great results in term of yield: phospholipase C hydrolyses phospholipids to release DAG, while other phospholipases and lipase, i.e., A1, A2, and B, hydrolyse the acyl groups from various lipid classes, facilitating transesterification reactions to obtain fatty acids methyl esters (FAMEs), which are the main constituent of biofuels (41). Compared with chemical approaches, the biocatalytic one gives a higher level of FAMEs without the use of excessive amounts of methanol, exclusively because of the kinetics and stereoselective properties of these enzymatic reactions (39). Biorefining, unlike a typical petrochemical refining process, is not based on a diminishing starting material but has the potential to be totally renewable and sustainable: solar energy capture, products diversity, harvesting, replantation of oilseeds, biomass waste conversion, and the use of enzymatic tools to purify the final products are the driving force for a new and sustainable source of fatty acids and biofuels entirely based upon bioeconomy (42).

## Marine Biome and Microalgae are Very Efficient Fatty Acids Bio-Factories

**Figure 3:**
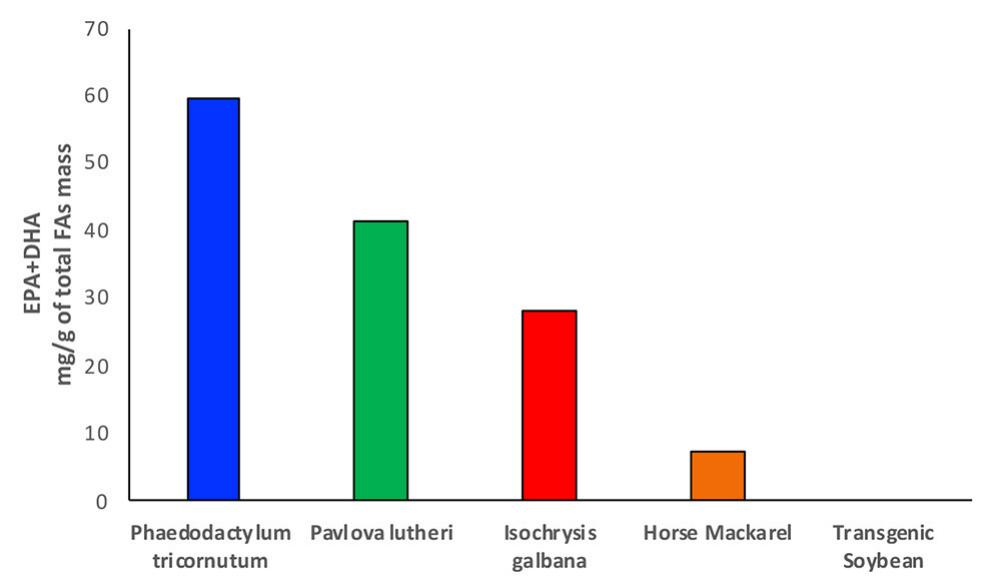
The bar chart shows the amounts of EPA and DHA produced in microalgae and compared with omega-3 oil rich fish and transgenic soybean plants. Data taken from Amjad Khan et al. (27) and Adarme-Vega et al. (53).

Until recently, fish has been considered one of the most valuable sources of fats and oils, especially for food applications, production of enriched fatty acid products and animal feedstocks (43). Fish and other marine sources, particularly salmons, mackerels, and mullets, which are rich in EPA and DHA, have been largely depleted, with a high risk to disappear within few years (44). Different studies have shown controversial results in terms of amounts and class of fatty acids present in farmed and wild fish when comparing the respective lipid profiles. The ratio between ω-3 and ω-6 fatty acids seems to vary from one species to another and according to the composition of fish feeds (45). Particularly, research on sea bass lipid content has shown how the farmed fish exhibits a higher level of omega-6 LA and a consequent decrease in the ω-3/ω-6 ratio compared with the wild fish, where the ratio has been found to be considerably higher (45). This effect has been attributed to a large amount of ω-6 fatty acids contained in fish feeds, which are in fact rich in terrestrial plant oils. Surprisingly, the opposite has been shown in another study between wild and farmed salmons, where the wild species seems to have a lower content of ω-3 instead despite having a much larger variety of food directly available from their native habitat (46). Another aspect that has to be considered is the amount of heavy metals and other contaminants that can be found in fish and marine sources extracts, which do not meet the health and nutritional security standards (47, 48). Fish and fish oils are, in fact, one of the primary sources of exposure to certain contaminants such as methylmercury, demonstrated to be highly neurotoxic, polychlorinated biphenyls, and many other persistent halogenated organic pollutants, whose content seems to be similar between farmed and wild fish (49). The problem related to the balance between the health benefit derived from the high value fatty acids pull and the level of toxic substances generates concerns around the usage of fish as primary source of fats (49). Furthermore, the constant increase in human population and in demand of food and energy sources, fish, and other marine sources clearly do not represent the mean to satisfy the current world supply of fatty acids (50). For all these reasons, fishing and, in particular, overfishing have been declining since 1980, and the interest in new sources from deep and shallow sea water has been growing (47, 51). For a long time, the focus on the study of lipid sources has been limited to deep water organisms such as fish, crustaceous, corals, and zooplankton, which are very rich in phospholipids, triacylglycerols, wax esters, and sterols, and therefore of the desired fatty acid building blocks. Marine microbiome is one of the richest sources of fatty acids and particularly of essential ω-3 PUFAs, such as EPA and DHA, which have been already mentioned in the previous paragraphs for their properties in human health (52) (Figure 3). Particularly, microalgae have one of the highest oil content, between 10 and 50%, and they are able to produce up to 30–70% of total lipid mass, with a very high percentage of this being EPA and DHA (54) (Figure 3).

**Figure 4:**
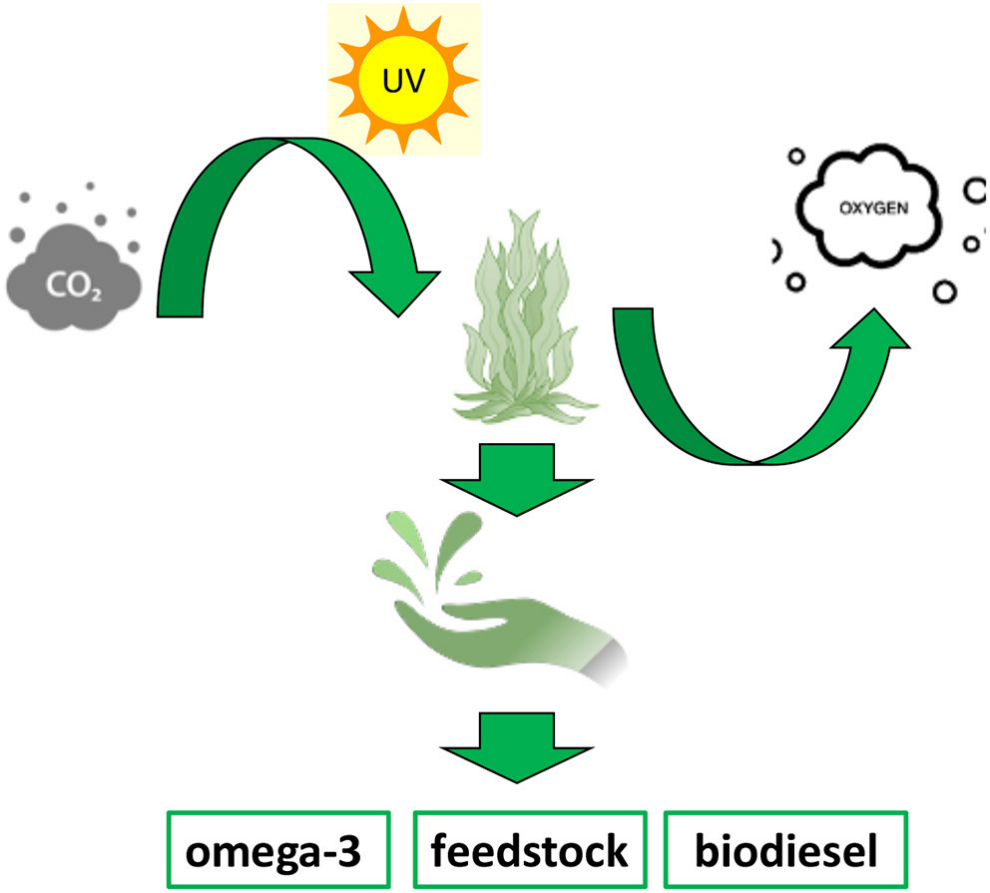
The cartoon is a schematic representation of the algae aquaculture bioprocess and of different products that can be obtained by fatty acid-rich biomasses.

These organisms are able to accumulate fatty acids for energy storage to be used for all their metabolic functions (54). In fact, microalgae vary the synthesis of different amounts of endogenous PUFAs and other metabolites *via* an adaptation process in response to altered levels of sea depth, temperature, UV light, and oxygen and carbon dioxide levels. This remarkable environmental response shows to be a promising opportunity to modulate, on demand, the amount of specific classes of fatty acids required while maintaining the equilibrium of the aquatic environment in a very finely regulated manner (53). One example of this extraordinary ability has been proved by the 2-fold increase in total lipids mass, obtained, respectively, in *Phaeodactylum tricornutum* (55), *Nannochloropsis spp*.(56), and *Dunaliella spp*. (57) by changing light intensity, salt concentration, and temperature. ω-3 fatty acid biosynthesis has also been modulated, determining an increase in EPA production of around 10%, at lower culturing temperature both in *Pavlova lutheri* (58) and *Phaeodactylum tricornutum* (59). More recently, genetic manipulation and transcriptional engineering have found a space in order to optimise the synthesis of high value fatty acid products from microalgae (60). In fact, according to their degree of unsaturation, microalgae oil content can be used for food supplements and pharmaceuticals or biofuels production. Therefore, a genetically modified and tuneable system showed to be a promising approach to improve the production of a desired degree of unsaturation in the final product, determining higher fatty acid diversity and efficient producing process (61). A way of implementing the amount of fatty acids has been exploited by slowing and reducing the process of fatty acids degradation by lowering the expression level of beta-oxidase genes and consequently their activity. Another example consists of the overexpression of elongases and desaturases, which have shown the possibility to tune and enhance the production of ω-3 VLC-PUFAs. A remarkable example of this approach is the overexpression of the elongase ELOVL5 in *Phaeodactylum tricornutum*, which resulted in a much higher percentage of DHA and EPA as final products. Moreover, it established the potential for a large-scale production of PUFAs from microalgae at industrial level (62). An analogous approach has been applied to the production of biofuels. In order to obtain fast-growing and oil-rich microalgae biomass, malic enzyme, involved in pyruvate metabolism and carbon fixation, from *Phaeodactylum tricornutum* has been overexpressed. This genetic modification determined an increase in the amount of neutral lipid of 2.5-fold and of 60% compared with the wild type. Thus, it suggested a potential new path for developing specifically designed microalgae strains to improve and facilitate the production of biofuel (63). Microalgae are also widely used as enriched feed stock source for aquaculture and animal farming. In fact, the products obtained from microalgae biomasses, either fresh or dried, are used as fatty acid-enriched source directly for livestock (Figure 4) (53). In this way, the use of omega-3 enriched microalgae biomass has been largely applied to indirectly increase the human consumption of essential PUFAs (43). In fact, the use of those as supplement to faming animals feed has resulted in increased concentration of EPA and DHA in milk, eggs, and meat, which are then addressed to human diet (64–66).

Furthermore, the microalgae biomass can be highly purified to obtain pure chemicals for pharmaceutical applications, such as the production of ω-3 food supplements (Figure 4). In fact, due to the benefit that humans are able to gain from a diet rich in EPA and DHA, and the fact the microalgae are the largest bio-producers of those, the products from refined biomasses have claimed a great role in many medical applications, such as for inflammatory chronic disease, allergies, cardiovascular disease, and neurological diseases among others. Recently, they have found an application in drug delivery as well: their very low cytotoxicity and high lipophilicity make them able to be used as a medium to facilitate the crossing of cell membranes of many active principles (67).

The flexibility and adaptability of microalgae and the fact that they are relatively easy to grow and genetically manipulate have defined these marine microorganisms as excellent bio-factories for valuable fatty acid products and by-products on the whole (Figure 4) (53, 61).

## Oleaginous Microorganisms and their Resourceful Lipid Storage System

**Figure 5:**
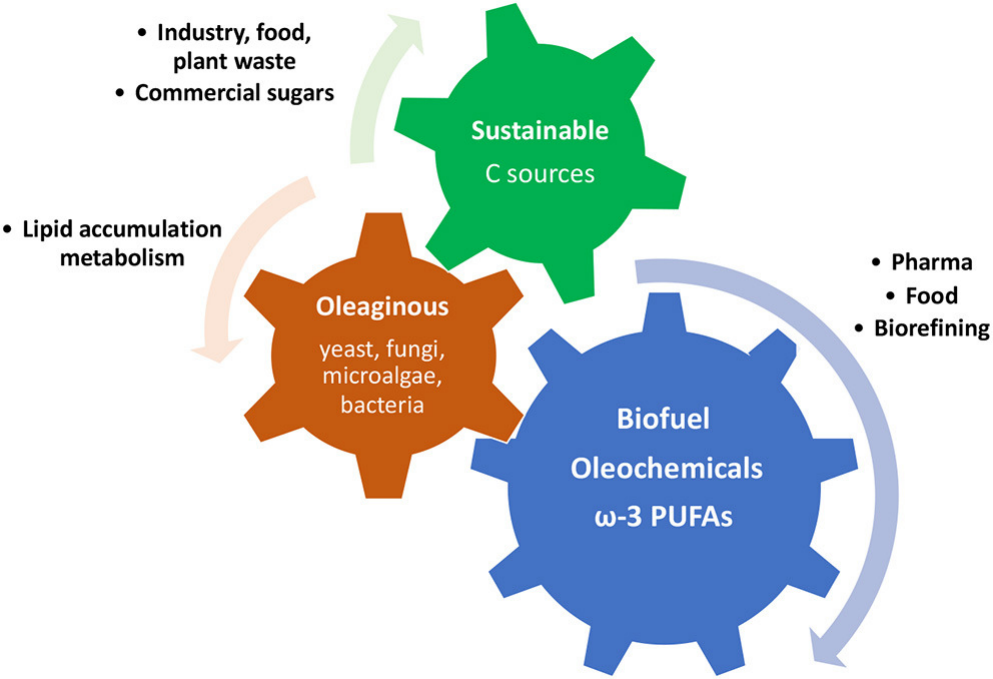
Schematic representation of the mechanism of production and utilisation of fatty acids and lipids produced by oleaginous microorganism using cost-effective carbon sources.

Some microorganisms, such as filamentous fungi, yeast, some microalgae, and some bacteria, are defined as an oleaginous microorganism because of their remarkable ability of storing intracellular lipids into lipid droplets, known as single cell oils (SCO), which are particularly rich in triacylglycerol (68). The level of lipids accumulated is usually between 20 and 80% on the total cell biomass, and it can vary according to culturing conditions as much as the fatty acid profile on the whole (68). The large amount of lipids produced and the diversity of fatty acid molecules that can be obtained through the production of SCOs are becoming more and more attractive in terms of finding alternative sources of fatty acids. They have been shown to be an advantageous alternative to plants, animals and fish oils and fats, especially for biodiesel production and for some ω-3 PUFA products (Figure 5). In fact, oleaginous microorganism cultures, compared with plant cultivation, animal farming, and aquaculture, do not depend on location, climate, seasons, and space. Moreover, they are able to use various carbon sources, from food industry waste to renewable carbon sources (54, 69).

Unlike other non-oleaginous microorganisms in the same conditions, in the presence of an excess of carbon and a lack of nitrogen, their growth rate is not slowed or stopped. Instead, their metabolic response decreases their total biomass and increases their lipid production (70). Many studies have shown that it is possible to reach a higher level of SCOs using very cheap carbon sources or industrial by-products, such as glycerol, commercial sugars, and plant and lignin materials (Table 1). The use of this approach proved that yeasts are able to accumulate up to 22% (w/w) of lipids and fungi up to 43% (w/w). Very successful is the application of even cheaper carbon sources from plant wastes, such as orange and tomato peels, obtaining a total lipid amount >50% in various oleaginous microorganism (72–74). Particularly, the oleaginous fungi *Cunninghamella echinulata* produced 46.6% of total lipids, such as 14% of gamma linolenic acid (75).

**Table 1:** The table shows few examples of some oleaginous microorganism cultures supplemented with cheap and waste feedstock carbon sources and the relative amount of lipids obtained.

**Oleaginous microorganism**	**Carbon source**	**Lipid content (w/w, %)**
*C. vulgaris* NIES-227	Glucose, low nitrogen	89
Auxenochiorella prototheconides	Birch biomass	66
Rhodosporidium kratochvilovae	Clarified butter sediment waste	70.74
Cryptococcus curvatus	Waste cooking oil	70
Rhodococcus opacus PD630	Biomass gasification	66
*Gordonia sp*. DG	Sunflower oil	52

*Data are selection of the highest lipid contents reported for some microorganism and collected in Patel et al. (71)*.

Another great advantage of this cost-effective source of fatty acids is that they can be cultured under solid state fermentation, obtaining an even higher productivity of SCOs with lower cost for media usage, and low energy and water consumption. The use of this sustainable carbon sources allowed the synthesis of high levels of PUFAs at lower cost, such as in *Mortierella sp*. (76). Particularly, ARA was produced up to 70% by *M. alpina*, whereas *M. hyalina* and *M. elongata* made up to 23% of ARA and higher concentration of oleic acid (77). The production PUFAs from oleaginous microorganism has found important application in food industry for food supplementation (78). One of the first oil obtained from *Mucor circinelloides* was commercialised in the 1980s as an alternative source of gamma-linolenic acid (GLA) from oil seeds (79). In the 1960s, oils rich in ARA started to be introduced in food industry and cosmetics. ARA and DHA rich SCOs obtained from *M. alpina* and *Pythium sp*. showed the highest yield obtained with this technique, and from 1985 were launched in the market for infant formulas such as milk, reaching a very high level of consumption around 2010 (80). Despite being a promising source of PUFAs, SCOs production has not found the predicted wide usage especially in food and pharmaceutical industry. There are, in fact, some disadvantages in the scale-up process and products recovery of SCOs (81). The complex procedure of extraction often requires application of toxic solvents, which is not allowed in food industry (82). On the other hand, the potential and applicability for biodiesel and oleochemicals seem more promising and advantageous (68). Oleaginous microorganism cultures have short life cycle, account for adaptability and independence from external factors; therefore, they have the potential to be applied for large-scale industrial production of biofuel, with reduced impact on energy consumption and exhaustion of soil (83). *Aspergillus niger* cultivated on sugar cane waste from distillery has been shown to be resourceful to produce biofuels from low-cost waste feedstocks (84). Although this approach looks promising and sustainable, there are still very high cost of downstream extraction and refining, that inevitably counteract with the relatively cheaper one used for plant and animal sources (85). Moreover, SCOs are also studied as potential source of oleochemicals such as fatty acids, fatty alcohols, and fatty acid methyl and ethyl esters as substitutes of the ones synthesised from petrochemical feedstock (86). Together with various supplementation approaches and vary cultivation techniques, a big improvement is given by the use of metabolomic and genetic tools such as overexpression and knock-down of specific genes that have a fundamental role in the lipids and fatty acids metabolisms (71). This approach has allowed a tuneable, selective and larger synthesis of endogenous lipids in *Yarrowia lipolytica*: the co-overexpression of the two key genes encoding foracetyl-CoA carboxylase (ACC1) and diacylglycerol O-acyl transferase 1 (DGAT1) led to a 5-fold increase in total lipids content (87). This oleaginous yeast has been considered one of the most promising platforms for metabolic engineering of FAs metabolism, followed by others such as *Lipomyces starkeyi, Cryptococcus curvatus*, and *Thriosporum pullulans*, which still need a more extensive study of metabolic pathways and genomic tools to design efficient cell factories (88).

Oleaginous microorganisms have shown an undoubted great potential to be green, sustainable, and genetically tuneable sources of fats, but the high cost estimated to grow large scale cultures still raises concerns about this being a more cost-effective process compared with the classic approaches. However, the utilisation of renewable carbon sources derived from wastes can partially reduce the problem. Furthermore, the introduction of those as alternative sources of biofuel helps to lower the overall production costs, as well as getting ω-3 PUFAs from more sustainable sources, without further depleting the already affected marine ecosystem (71).

## Biotechnological Advances, Synthetic Biology, and Mathematical Modelling to Build a Cell Factory for High Value Fatty Acids Products

**Figure 6:**
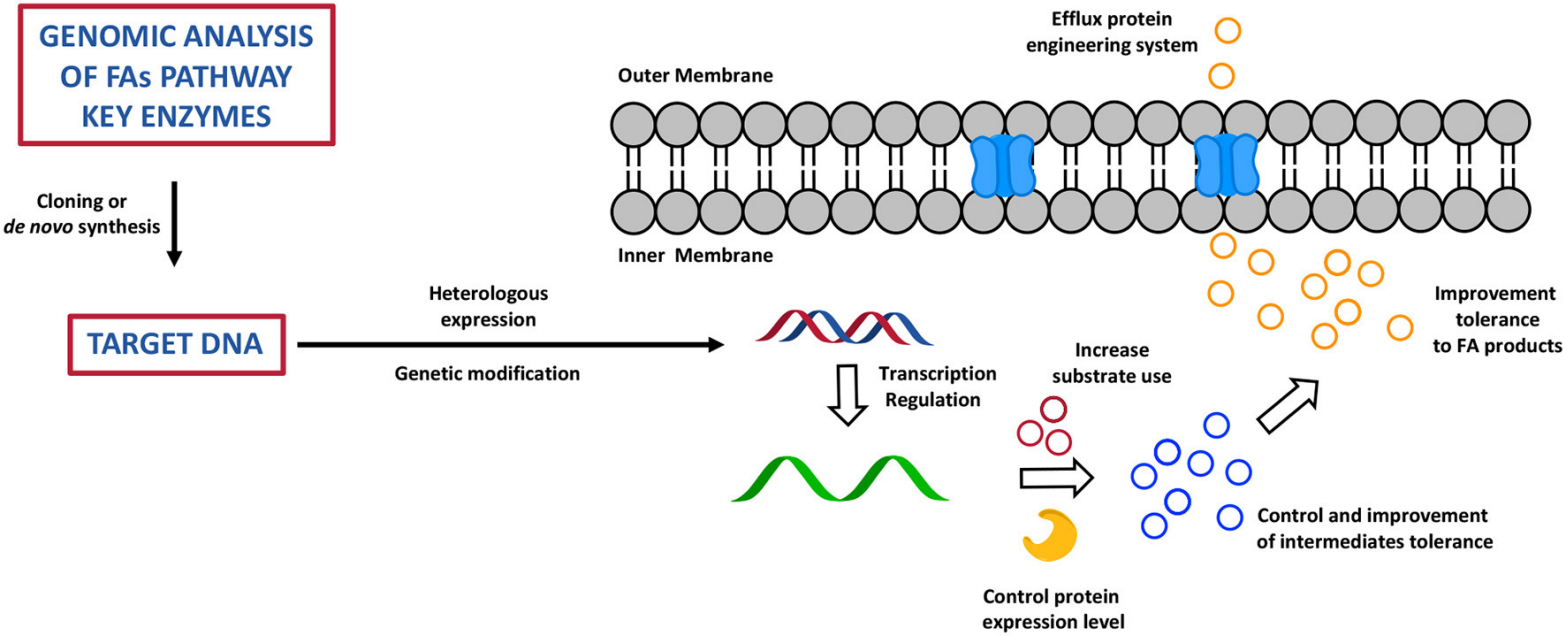
The cartoon represents the most important step of the bioengineering process of cell factory to produce high valuable fatty acid products. This has been adapted from Yu et al. (93).

Chemical biology and metabolic engineering represent the new frontier to improve the productivity of a broad range of organisms in order to find the most efficient way to overcome the shortage of FAs sources and to specifically tune the synthesis of certain species rather than others (86). This approach has also shown to be able to overcome a series of disadvantages derived from the usage of natural sources of fats, i.e., fish and plants, as well as the often non-economically viable and time-consuming chemical processes of FA derivatives total synthesis or extractions from microorganisms (89). Today, this approach is led by broad knowledge around synthetic enzymatic pathways of FAs, PUFAs, and FA derivatives (FA alka(e)nes, FA alcohols, FAMEs, and fatty acid ethyl esters (FAEEs) and by a wide variety of biochemical and genetic tools that researchers have available to design cell factories to produce high value fatty acid products (90). The idea is based upon reconstruction of the FA biochemical pathway through a fine regulation and manipulation of gene expression, through deletion, overexpression, or lower expression of one or multiple genes that encodes for key enzymes, which ultimately results in a highly controlled phenotype, and therefore in effective production of the desired metabolic products (71). Genomics, metabolomics, transcriptomics, proteomics, high-throughput screening, and computational studies have been largely applied to predict and optimise the design of the cell factories in order to achieve the best result possible (71, 86). As discussed before, different types of microorganism are able to produce and accumulate fatty acids and their derivatives, so how do researchers choose their cell factories? In order to maximise the efficiency of a modified system for production of fatty acids, it is fundamental to choose a microorganism that is widely studied and used in metabolic engineering. Therefore, the heterologous systems of choice are usually *S. cerevisiae, E. coli*, and few others: they have a high bio-safety score, they are easier to generically manipulate, they are fast and cheap to grow at high cell density, they rapidly adapt to different conditions (temperature, pH, carbon sources, etc.), and therefore they have been considered industrially relevant and viable (91, 92). Many studies have been carried out to prove that it is possible to increase and facilitate the otherwise complex and, in some cases, expensive production of FAs (93). The first step in this process consists of the identification of sequences of genes that encode for enzymes that have a primary role in the FA synthetic pathway and that are very efficient in certain microorganisms (93) (Figure 6). These genetic sequences are then cloned or *de novo* synthesised to be finally introduced *via* recombination processes, with potential codon optimisation and/or targeted genetic mutations into the DNA of an heterologous system (93) (Figure 6). At this point, the engineered host microorganisms are able to start a process of transcription, which can be also modified to control the level of genetic expression *via* inducible promoters and terminators, and the level of RNA, controlling its folding and degradation through biological sensors, in the heterologous system: this dynamic and fine control of the transcription process has shown to be an excellent tool to improve the yield of FA products and to be particularly useful in the production of biofuels (94, 95) (Figure 6). At this point, once transcription is completed, translational engineering tools can be used to vary the yield of the enzyme produced and to lower its degradation and also the speed at which it is synthesised (93) (Figure 6). Post-translational modifications (glycosylation, phosphorylation, methylation, acetylation, ubiquitination, proteolysis) of the enzyme are applied to improve the affinity of the active site for the substrate and therefore the final FA product yield. In order to avoid cell toxicity, the control and the improvement of the level of tolerance for the intermediates and for the high level of the final fatty acid products is important (93) (Figure 6). The very final step is based on engineered systems for the efflux of fatty acids from the cell. This is a very new tool which is still under investigation (93) (Figure 6).

Following this complex and fine approach, many different high value fatty acid products have been obtained in high yield in both *E. coli* and *S. cerevisiae*. A great example of designing and engineering an FA biosynthetic pathway, which takes in account the tolerance and toxic effect of the overproduction of the final metabolite, is the synthesis of gamma-hydroxy fatty acids (96). This class of molecules is of great interest, because they give long carbon chain monomers that can be used in the synthesis of polymer materials and as building blocks or intermediates in chemical, pharmaceutical, and food industries (96). In this study, *E. coli* has been chosen as heterologous system for the expression of an alcohol dehydrogenase (ADH) from *Micrococcus luteus*, a monooxygenase (BMVO) from *Pseudomonas putida* KT2440, and an esterase (PFE1) from *Pseudomonas fluorescens*. This engineered biosynthetic cascade produced gamma-HUA at a productivity of 3.2 mM/h and more than 80% yield (96). *E. coli* has also been used successfully to produce biofuel as an alternative to the transesterification of triacylglycerols (TAGs) extracted prevalently from oil plant seeds (97). This study showed a scalable and sustainable *de novo* biosynthesis of FAEEs from glucose, obtained from lignocellulose biomass, into a genetically modified *E.coli* to exploit the ethanol-producing pathway from *Zymomonas mobilis*, to increase the fatty acyl-CoA pool and the heterologous expression of acyl-CoA:diacylglycerol acyltransferase from *Acinetobacter baylyi* (97). The total amount of FAEEs (particularly ethyl palmitate, oleate, myristate, and palmitoleate) reached a level of 922 mg/L (97). An approach that seems to provide a good, sustainable, and stable strategy for a large-scale production of VLC-FA-derived chemicals has been presented in a study that used *S. cerevisiae* as heterologous system (98). This engineered platform has been designed to express the fatty acid synthase I (FAS I) from *Mycobacterium vaccae* and a specific fatty acid reductase (C22), obtaining selectively 83.5 mg/ of docosanol, which is very useful for chemical and biofuel production (98). Looking at the synthesis of essential FAs, a great majority of microorganisms have a low capacity to metabolise and accumulate PUFAs and especially omega-3 and omega-6 in their cell systems. Therefore, these biotechnological advances and genetic manipulations have been applied to tune PUFA production by the construction of an effective and now widely used synthetic metabolic pathway. An example is the heterologous expression of desaturases and elongases from different oleaginous microbes for production of industrially relevant ω-3 and ω-6 PUFAs by up to 35% in the fungus *Ashbya gossypii* (99).

In another recent study, the polyketide synthase-like PUFA from *Mycobacteria* was reconstructed in *Y. lipolytica*, overcoming the traditional system of elongases and desaturases normally preferred, and resulting in a highly enriched lipid profile with a promisingly high yield of PUFAs (100). These are only few examples of a very innovative platform of investigations on alternative and sustainable sources of fatty acids, which has shown to be very promising. It suggested that metabolic engineering of FA metabolism at different levels and the use of biotechnology could be one of the best approaches to increase the amounts of available FAs. Alongside this, synthetic biology has been shown to offer very attractive and promising roots to design novel biological cell factories to increase fatty acid and oil production (101). In fact, it has been possible to reproduce exact biological behaviour and metabolic pathway for fatty acid and lipid synthesis with high fidelity by assembling the natural components of a specific cell system (such as proteins, enzymes, and organelles) and chemically synthesised molecules to mimic substrates and products (102). In order to enable an optimised reconstruction of these extremely complex biocatalytic systems, computational, and mathematical modelling have been widely and successfully introduced in synthetic biology (101). The *in silico* mathematical simulations and calculations represent powerful tools to predict the dynamics of a metabolic cascade and to search a very large space of physical and chemical parameters, aiming to exclude unfavourable conditions and promote an ideal environment, finally achieving the desired response into a bioengineered system (103). One of the most representative examples of the use of this method is the production of biofuels and oleochemicals feedstocks from oleaginous microorganisms (104). In particular, scientists have been able to provide a system-level view from a large range of these organisms by bringing together top-down and bottom-up approaches (104). The first one is based on genomics, metabolomics, proteomics, and transcriptomics high-throughput screening and accurate data collection from the SCO cellular environment (105, 106). The second one relies on mathematical predictive models, which are developed on pre-existing knowledge around SCOs, in order to perform a systematic analysis of the cellular environment during the biochemical switch from lipogenesis and lipid accumulation (107, 108). These two methods have successfully given a full understanding of lipogenesis in SCOs by highlighting a similar metabolic pattern across all microorganism studied, and by finally leading to the identification of key regulatory hotspots such as glucose-6-phosphate dehydrogenase (G6PDH) (109), 6-phosphogluconate dehydrogenase, malic enzyme (ME) (110), and ATP citrate lyase (ACL) (110), which are all promising targets for bioengineering the synthesis of lipids, in order to obtain an optimal amount of lipid for oleochemical production (108, 111).

## The Role of Fatty Acids in Biorefinery: the Challenge of Environmental Impact and Circular Economy

**Figure 7:**
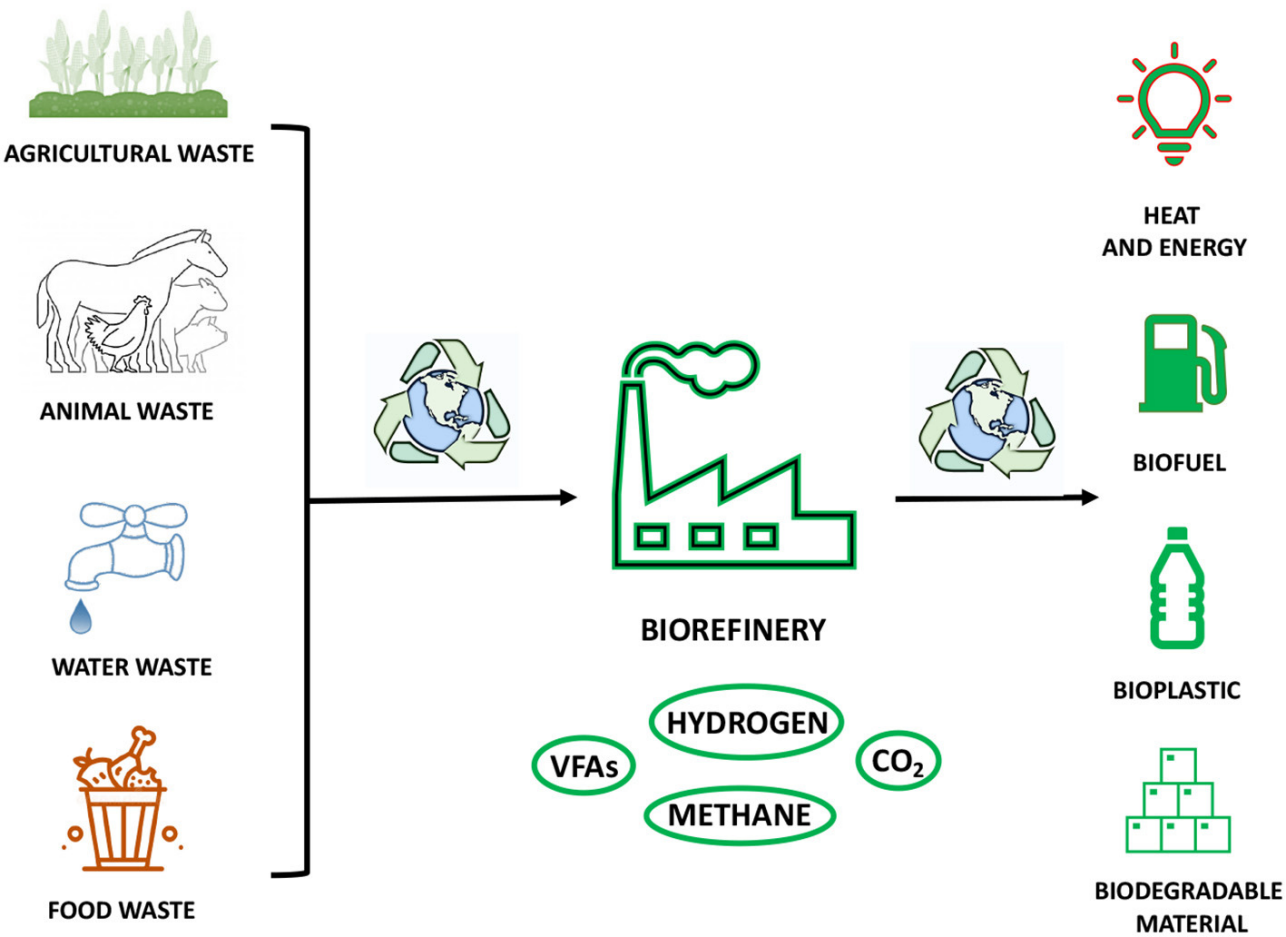
The cartoon is a representation of the use of organic waste in the biorefinery process to obtain valuable products in a sustainable and environmentally friendly manner.

The economic, social, and technological development of the modern society has come into place together with corresponding very high consumption and demand of both renewable and non-renewable resources. The immediate consequence is an acute impact on the environment in terms of waste of resources at the “end of their life,” pollution, and global temperature warming (112). As discussed throughout this review, the production of FAs can be considered as an emblematic example of scientific effort to maximise the yield and conversion of FA building blocks into high value products by embracing a more sustainable and environmentally friendly use of resources (113). At this regard, FAs, and, in particular, volatile fatty acids (VFAs), have gained an important role in the emergent concept of biorefinery (Figure 7) (114). This innovative approach is entirely based on the usage of organic wastes, such as food and landfill wastes, and other renewable sources, such as animal feedstock, farm biomasses, and industrial waters, in order to produce an added high value, biopolymers, biogases, and biofuels (Figure 7) (115). The recovery of waste resources, which would otherwise be disposed of *via* incineration, with consequent emission of high level of toxic greenhouse gases and chemicals, would allow the capture and reutilisation of carbon sources in a more efficient manner (Figure 7) (116). Moreover, this approach would offer the chance to replace fossil fuels, which are largely used today (116). The production of VFAs is an outstanding example of how sustainable approaches and biotechnologies can be applied in large-scale production to obtain raw materials by recycling organic wastes in a very effective way (Figure 7) (117). These compounds have a great value in chemical industries, because they can be applied as substrates for biofuels, such as methane and hydrogen, and for biopolymers, such as polyhydroxyalkanoates (PHA) and biodegradable plastics (Figure 7) (114, 118). In order to obtain VFA building blocks, feedstock, food, and landfill wastes are used as alternative and rich sources of organic matter, containing high levels of C, N, and P (119, 120). Thus, the classic oxidation and carboxylation of aldehydes and alkenes obtained from fossil fuels could be replaced by biofermentation techniques. This biotechnological approach relies on mixed anaerobic bacterial cultures and their various metabolic pathways, which depend only on proteins, carbohydrates, and lipids as macromolecular substrates (121, 122). Therefore, from the fermentative breakdown of a complex waste organic matter, it is possible to obtain amino acids, sugars, FAs, and glycerol, which are then transformed through acidogenesis into VFAs and other fermentation products, to finally give hydrogen and carbon dioxide, which can be further used in polymerization processes, and methane (Figure 7) (123). The entire biorefinery cascade shows to be an efficient and powerful system to yield various building blocks for biodegradable plastic-like materials and biogases (Figure 7) (114). Thus, it has the potential to drive industry of plastic on an alternative route by favouring the production of biodegradable materials at the expense of petroleum derivatives (124). Moreover, the rich pool of biogases, such as hydrogen and methane, obtained during biofermentation, accounts for all the requirements to be alternative, sustainable, renewable, and low-cost sources of energy: the fuels of the future, as they have been recently defined, can become the most valid alternatives to fossil fuels as source of bioenergy for households, cars, and industries (Figure 7) (125). The utilisation of agricultural waste, industrial waste, and municipal organic waste to obtain FA building blocks and high-value derivatives, is just one of the many examples that the modern and industrialised world should take into consideration to help to slow down and reduce global warming and overall climate change (Figure 7).

Undoubtedly, the approach proposed for alternative FA production has accepted the challenge proposed by the new model of circular bioeconomy: recycling and reuse are the foundations for designing and optimising various production streams, while still promoting economic growth, but with the advantage of reduced environmental impacts (126, 127). Nevertheless, this new approach is still suffering from some limitations, such as assigned budgets, infrastructure adequacy, improper treatment of waste resources, in both developed and developing countries (128). Inevitably, the adaption to this bioeconomic productive system comes out with costs, and many questions have been raised especially around the extensive use of land required (129). Notwithstanding the impressive steps forward made by the previously discussed biotechnologies in the field for production of high-value chemicals, such as FAs, there is still a large slice of the production that relies totally on use of considerable amount of farmland for crops and feedstock productions (130). This disadvantage brings up a fundamental ethical debate: is it acceptable to exploit land that could be used to increase the production of various indispensable food resources for many developing countries, almost exclusively for industrial purposes? How can the concept of circular bioeconomy meet this essential requirement while trying to overcome global environmental impacts? Unfortunately, the questions have not been answered yet, and they have highlighted one of the greatest contemporary scientific and socio-economic challenges of history, not only for the FA industry: finding a new production strategy that allows social, economic, and environmental planet health at the same time. One of the possible ways forward into this challenging but also inspiring path is to propose a multidisciplinary, low-cost, and sustainable approach that allows broad knowledge of different fields to come together for the same aim, increasing the chances of successful discovery.

## Conclusion and Future Perspective

Fatty acids are one of the major constituents of all organisms, and they play essential structural and functional roles for the biology of cells. As it has been underlined in this review, FAs and their derivatives have extended values that go beyond their biological properties: they are building blocks for a large variety of chemicals that can be applied as high value starting materials in various industrial fields such as food, feedstocks, pharmaceuticals, cosmetics, biorefineries, plastics, oleochemicals, and many others. It is also clear that PUFAs are essential for human health and for prevention of chronic diseases. The biggest issue encountered so far is obtaining a sufficient level of FAs and PUFAs in order to meet the continuously increasing world demand for those, taking into account the modern world challenge of looking for sustainable, renewable, and cost-effective sources. Much progress has been made in terms of reinventing and rethinking the way in which fatty acids sources are identified and used. Nowadays, in fact, with the advent of green chemistry, climate change, protection of the environment, and bio economy, scientists have been looking into alternative ways of obtaining FAs in large amount, without damaging the marine ecosystem and vegetation. It has been indeed a challenge, which has seen the discovery of many bio-technological advances along with broader knowledge of the metabolism of PUFAs and FAs and their derivatives in a wide number of organisms such as microalgae, yeast, fungi, bacteria, and plants. Microorganisms are shown to be remarkable producers of fats: chemical biology investigation, and genetic and metabolic engineering have become very advanced and efficient tools for researchers to obtain a high yield of fats and lipids. The approach to this innovative method has been also allowed by exploiting the advantageous fast growth, adaptability, and allowance for genetic manipulation of these microorganisms. Thus, a new path to obtain highly sought-after/high-value FAs has been marked with a renewable, green, and low-cost aspect: design of metabolic engineered cell factories is the way forward to overcome the lack of FAs, to improve not only the yield and chemical variety but also the potential for large-scale production. This is just the beginning of a very promising and even more advanced strategy that fatty acid biotechnology will see in the near future. In order to achieve this goal, more scientific efforts are required to reach the widespread applicability of this newest methodology that will eventually enter industrial production and finally commercialisation.

## Author Contributions

TKS came up with the idea and reviewed it. MC wrote the article.

## Conflict of Interest

The authors declare that the research was conducted in the absence of any commercial or financial relationships that could be construed as a potential conflict of interest.

## Abbreviations

ACC1: Acetyl-CoA carboxylase
ADH: Alcohol dehydrogenase
ALA: Alpha-linolenic acid
ARA: Arachidonic acid
BC: Before Christ
DAG: Diacylglycerol
DGAT1: Diacylglycerol O-acyltransferase 1
DHA: Docosahexaenoic acid
EPA: Eicosapentaenoic acid
FA: Fatty acid
FAEE: Fatty acid ethyl ester
FAME: Fatty acid methyl ester
FAS I f: Fatty acid synthase I
LA: Linoleic acid
MUFA: Monounsaturated fatty acid
OA: Oleic acid
PA: Palmitic acid
PUFA: Polyunsaturated fatty acid
SA: Stearic acid
SAFA: Saturated fatty acid
SCO: Single cell oil
TAG: Triacylglycerols
UV: Ultraviolet
VFA: Volatile fatty acids
VLC-PUFA: Very long chain polyunsaturated fatty acids.
